# Piezoelectric Templates – New Views on Biomineralization and Biomimetics

**DOI:** 10.1038/srep26518

**Published:** 2016-05-23

**Authors:** Nina Stitz, Sabine Eiben, Petia Atanasova, Neus Domingo, Andreas Leineweber, Zaklina Burghard, Joachim Bill

**Affiliations:** 1 Institute for Materials Science, University Stuttgart, Heisenbergstr. 3, 70569 Stuttgart, Germany; 2 Institute of Biomaterials and Biological Systems, University Stuttgart, Pfaffenwaldring 57, 70569 Stuttgart, Germany; 3 Oxide Nanoelectronics Group, Catalan Institute of Nanoscience and Nanotechnology, ICN2 Campus de la UAB, 08193 Bellaterra, Barcelona, Spain; 4 Max Planck Institute for Intelligent Systems, Heisenbergstr. 3, 70569 Stuttgart, Germany

## Abstract

Biomineralization in general is based on electrostatic interactions and molecular recognition of organic and inorganic phases. These principles of biomineralization have also been utilized and transferred to bio-inspired synthesis of functional materials during the past decades. Proteins involved in both, biomineralization and bio-inspired processes, are often piezoelectric due to their dipolar character hinting to the impact of a template’s piezoelectricity on mineralization processes. However, the piezoelectric contribution on the mineralization process and especially the interaction of organic and inorganic phases is hardly considered so far. We herein report the successful use of the intrinsic piezoelectric properties of tobacco mosaic virus (TMV) to synthesize piezoelectric ZnO. Such films show a two-fold increase of the piezoelectric coefficient up to 7.2 pm V^−1^ compared to films synthesized on non-piezoelectric templates. By utilizing the intrinsic piezoelectricity of a biotemplate, we thus established a novel synthesis pathway towards functional materials, which sheds light on the whole field of biomimetics. The obtained results are of even broader and general interest since they are providing a new, more comprehensive insight into the mechanisms involved into biomineralization in living nature.

There are many examples for biomineralization processes in living nature leading to sophisticated functional materials, such as nacre, teeth or bones^1^,^2^,^3^. Biopolymers, such as proteins, guide the mineralization of the inorganic component by cellular control e.g. in vesicles. The main interaction mechanisms between the organic template and the inorganic phase are driven by electrostatic interactions and molecular recognition. It is known that many hierarchically organized biological macromolecules, such as deoxyribonucleic acid (DNA), and also proteins involved in biomineralization processes, are often piezoelectric. Despite this common ground, the role of piezoelectric contributions as additional interaction mechanisms was so far hardly considered, albeit, the influence of collagen proteins on bone formation has been proven. Ever since the discovery of collagen’s piezoelectricity in the 1960s, it was proposed as a potential mechanism for osteoblasts to detect areas of greater stress^4^,^5^,^6^,^7^,^8^ hinting to the impact of a template’s piezoelectricity on mineralization.

Some viruses also possess the structural requirements for piezoelectricity. Virus templates such as tobacco mosaic virus (TMV)^9^ and M13 phages^10^ exhibit themselves electromechanical properties due to dipole formation within the protein structure, giving high effective piezoelectric response. The origin of the radial effective piezoelectric coefficient in these viruses arises from the non-centrosymmetric structure of the polar protein in the axial plane of the virus fibers. In addition, due to their low stiffness, flexoelectric effects can also contribute to global piezoresponse as it was reported for single TMV via piezoresponse force microscopy (PFM) measurements^9^, for which the piezoelectric constant *d*_*33*_ was found to be as high as 12 pm V^−1^. Self-assembled thin films of M13 phages with enhanced intrinsic dipole moment by genetic engineering can exhibit piezoelectric strengths of up to 7.8 pm V^−1^ allowing successful integration of M13 phages in a virus-based piezoelectric generator^10^. Those values are considerably higher than the determined 1 pm V^−1^ of collagen^11^. Furthermore, it is reported that surface interactions of TMV and hydrophilic oxidized silicon lead to significant deformation of the virus structure^12^,^13^. The resulting mechanical deformation of the piezoelectric material can induce a voltage, which plays an active roles in synthesis processes. Additionally, TMV has excellent stability in a broad temperature and pH range in several organic solvents^14^. The virus can be genetically modified and allows the formation of homogeneous virus monolayers over large areas serving as templates for thin film formation^15^.

Inspired by nature’s biomineralization processes, low-temperature methods for synthesis of metal oxides like chemical bath deposition have been successfully combined with bio-templates such as DNA^16^ or TMV to prepare heterostructures for nanodevices to implement in field-effect transistors^17^. In particular, ZnO nanocrystallites are formed in solution by homogeneous nucleation and can interact with a template leading to film formation. The nanocrystallites are polar with an intrinsic dipole moment due to their non-centrosymmetric crystal structure and polar faces, namely a positively charged Zn-terminated and a negatively charged O-terminated plane.

ZnO has been brought into the focus of research due to the high electromechanical coupling factor and more specifically its piezoelectric coefficient, since piezoelectricity provides a direct conversion between mechanical and electrical energy and thus opens up the possibility of using these materials for sensing, actuation or energy harvesting applications. Several investigations have been performed on both, nanostructures such as nanowires^18^,^19^,^20^, and thin films^21^,^22^,^23^. As, a result, ZnO nanowires and nanobelts have also been suggested for applications in piezoelectric nanogenerators^24^.

In our work, biomimetic mineralization is achieved by the mechanical deformation of the TMV structure. This traces back to the strong surface interaction of TMV with the hydrophilic preoxidized silicon substrate, leading to a reduced height, larger width and also a reduced curvature compared to TMV particles in solution (Fig. 1a,b). The atomic force microscopy (AFM) topographic measurements of TMV particles immobilized on silicon confirmed this deformation. The AFM height image is given in Fig. 1c and the obtained average height over the virus length is given in Fig. 1d (procedure described in detail in the Supplementary Information). Analysis of the profiles showed strongly reduced height of 14 nm (Fig. 1d) compared to the nominal 18 nm of free virus particles, revealing a strong mechanical deformation of the virus cross-section in good accordance with published results^12^,^25^. In order to produce piezoelectric thin films, homogeneous TMV monolayers (Fig. 2) were produced via convective assembly^15^.

The deformation of TMV on hydrophilic silicon results in i) a strain of more than 22% on the axial plane of the virus, which can lead to a considerable enhancement of its piezoelectric properties due to increased parallel arrangement of the coat protein’s intrinsic dipoles after mechanical deformation (Fig. 3a), and ii) a reduced surface curvature, which improves crystal growth on top of it. Finally, the immobilized TMV particles undergo an additional reduction of the cross-section from 14 nm to 9 nm when immersed in the methanol reaction solution at 60 °C.

Ordered homogeneous monolayers of TMV on silicon substrates as shown schematically in Fig. 3b obtained via convective assembly technique (cf. Fig. 2) were used as template layers for ZnO mineralization processes. The monolayers were mineralized in methanol reaction solution resulting in growth of thin ZnO films (Fig. 3c). The mechanical deformation of the piezoelectric virus particles induces a voltage due to the piezoelectric effect attracting the polar ZnO particles, formed by homogeneous nucleation in solution, via dipole/dipole interactions while the flattened virus structure favors an ordered deposition. This ordered first deposited layer of ZnO crystals shown in Fig. 3b preserves a high polarity for the continued growth of the next ZnO layer resulting in a strongly pronounced *c*-axis texture of the homogeneous ZnO film (Fig. 3c) formed on the TMV template. This is revealed by X-ray diffraction (XRD), which shows a drastically increased relative intensity of the 002 reflection compared to the 100 and 101 reflections (Fig. 3d). As references for the mineralization process amino-functionalized (NH_2_-) and carboxyl-functionalized (COOH-) self-assembled monolayers (SAMs) were used as template layers. They do not exhibit piezoelectric properties, but both have functional groups that are present on TMV’s surface, hence, making accessible the biomimetic piezoelectric effect contribution. As shown by XRD, the distinct texture observed for the ZnO films deposited on the TMV template is not observed in the case of the assembly of ZnO crystals on the COOH and NH_2_-terminated SAMs (Fig. 3d) underlining the important role of the piezoelectric TMV template with regard to the textured growth of the ZnO films. The quantitative comparison of the degree of texture is given in the Supplementary Information.

Electrostatic contributions which may overcompensate the piezoelectric effect of the TMV could be ruled out by zeta potential measurements (cf. Supplementary Information, Figure S1). This is also supported by the fact that in solvents with low dielectric constants, in comparison to water, such as methanol (ε = 33.6) the capability of a solvent to electrolytically dissociate functional groups is strongly reduced. This is reflected by the higher dissociation constants, p*K*_a_ values, in methanol compared to water^26^,^27^. The higher the p*K*_a_, the lower the dissociation of the material is expected. For instance, the p*K*_a_ of carboxylic species, such as butanoic acid is increased from 4.82 in water to 9.69 in methanol^27^. Thus, the equilibrium is strongly shifted to the undissociated, uncharged species in methanol. Consequently, neither the COOH- and NH_2_-SAMs nor the TMV particles are expected to be charged in the methanol conditions and the deposition is not driven by electrostatic interactions. Accordingly, within the methanol reaction solution the dipole/dipole interactions of piezoelectric TMV and polar ZnO dominate deposition.

The piezoelectric properties were evaluated by PFM measurements performed on different samples. The piezoelectric coefficient *d*_*33*_ is generally defined considering that the piezoelectric response is measured using a capacitor-like structure in which the material is placed in a homogeneous electrical field. However, in the case of the PFM measurements, the field emerging from the tip is rather that of a point charge than a planar field^28^. Due to the non-uniform electric field emanating from the tip of the AFM during PFM measurements, determination of the *d*_*33*_ coefficient is not straight-forward. For the weak indentation regime, the *d*_*33*_ value can be approximated by *d*_*33*_ = 2 · *d*_*eff*_^10^,^29^. Moreover, the effective voltage sensed by the material will be smaller for a thicker sample due to the more radial electric field over the volume leading to a decreased effective *d*_*33*_ (cf. Supplementary information, Figure S2). In a proof-of-principle experiment ZnO films with differently tuned textures resulting in 100 and 002 textured films were synthesized. The results confirmed that only 002 textured films show piezoelectric behavior (cf. Supplementary information, Figure S3).

Piezoresponse of ZnO_002_/TMV samples with enhanced texture due to the piezoelectric effect of the TMV template during mineralization were compared to ZnO_002_/NH_2_ and ZnO_002_/COOH samples. All samples were mineralized applying 20 deposition cycles to avoid the above mentioned increased inhomogeneity of the electric field. The amplitude PFM images at 10 V for all samples are shown in Fig. 4a (above: ZnO_002_/NH_2_, middle: ZnO_002_/COOH and bottom: ZnO_002_/TMV). In the case of ZnO_002_/NH_2_ and ZnO_002_/COOH films, pronounced bright and dark areas can be seen, whereas in the case of ZnO_002_/TMV bright areas are dominant over a brownish background signal. This is affirmed by the amplitude profiles (Fig. 4b) which were taken along the grey lines in Fig. 4a. The response of the ZnO_002_/NH_2_ film shows deviations around a mean value of about 17 ± 9 pm (dashed line). The ZnO_002_/COOH film has a slightly higher mean response of 25 ± 12 pm. The maximum amplitude reached in both cases is around 35–40 pm. Finally, the ZnO_002_/TMV films show the highest mean value (55 ± 14 pm) and areas with increased amplitude of up to 86 pm. The strong deviations from the mean value arise from different facts. First, the piezoresponse highly depends on the electromechanical coupling between the sample and the AFM tip, which is strongly influenced by the roughness and topography of the sample. Second, the granular structure of the films may affect both, the homogeneity of the electric field in the *z*-direction, as well as it may produce incoherent coupling between the electromechanical response among different grains in *z*-axis, thus affecting the global effective signal. In order to account for all these effects, the amplitude signal was averaged over the entire image area and measured on five different places for each applied bias. The resulting curves are given in Fig. 4c. For all samples, a linear slope of the amplitude with increasing applied bias can be observed, corresponding to an experimental *d*_*eff*_ which is smaller than the real *d*_*33*_coefficient^30^.

The effective piezoelectric response, obtained from the linear slope in Fig. 3c, is doubled in the case of ZnO_002_/TMV resulting in a *d*_*eff,ZnO/TMV*_ = 3.8 pm V^−1^ compared to *d*_*eff,ZnO/NH*_*2*__ = 1.6 pm V^−1^ and *d*_*eff,ZnO/COOH*_ = 2.0 pm V^−1^. The piezoelectric virus template strongly enhances the texture due to dipole/dipole interactions leading to a high electromechanical response of the sample that can be estimated as *d*_*33*,ZnO/TMV_ = 7.6 pm V^−1^.

In summary, we successfully used the intrinsic piezoelectricity of a template for the synthesis of functional thin films with enhanced texture using a new biomimetic mineralization process. The mechanical deformation of TMV on oxidized silicon and additionally after immersion in the methanol reaction solution results in a flattened virus structure and induces a piezoelectric effect. This allows the well-ordered deposition of the polar ZnO crystals leading to a strong enhancement of the 002 texture together with a two-fold increase of the ZnO electromechanical response. The herein presented principle of using a piezoelectric template for the synthesis of functional materials can be extended to other templates and deposited functional materials. We were able to verify an additional property of the biotemplate significantly contributing to biomineralization processes besides electrostatic interactions and molecular recognition – the template’s piezoelectricity. The findings in this paper provide a new view on the general impact of a template’s piezoelectricity in biomineralization and opens up novel opportunities for the biomimetic synthesis of functional materials.

## Methods

### Self-assembled monolayer formation

Silicon wafers were modified with amino-functionalized (NH_2_) and carboxyl-functionalized (COOH) self-assembled monolayers (SAMs), respectively. The procedures are described elsewhere in detail^31^,^32^. For amino termination, a 2% (v/v) solution of 3-aminopropyltriethoxysilane (APTES) in 95:5 ethanol:water was prepared. Prior to immersion of cleaned silicon substrates (see cleaning procedure in Supplementary Information), the silane was left to hydrolyze for 5 minutes. After 60 minutes, the substrates were removed and thoroughly rinsed with ethanol and dried under N_2_.

For carboxyl termination, cleaned silicon wafers were immersed in triethoxysilylpropyl succinic anhydride (TESPSA; 10% in toluene) for 16h and subsequently sonicated in toluene, N,N-dimethylformamide (DMF) and nanopure water (20 minutes each) before drying under an N_2_ stream.

#### Virus monolayer formation

Uniform virus monolayers were obtained via convective assembly as described in detail in reference^15^. Experiments were performed in a self-made set-up in a glass chamber providing constant temperature (23 ± 2 °C) with controlled humidity triggered by a saturated LiCl solution (35 ± 2% relative humidity). The substrate was horizontally positioned on a sample holder connected to a computer controlled linear motor (KSV Instruments, Espoo, Finland) ensuring continuous motion with constant withdrawal velocity. A glass slide was fixed under constant angle. A droplet of the buffer-free virus solution was trapped between the silicon substrate and the glass slide forming a triple interface between virus solution, silicon substrate and air. Subsequently, the motor pulls with constant velocity stretching the formed meniscus over the surface. Upon solvent evaporation, a homogeneous dense virus layer is formed. The experiment was carried out using a virus solution with a concentration of 5 mg ml^−1^ and a withdrawal velocity of 1.2 mm min^−1^.

### ZnO Mineralization

Methanol stock solutions of zinc acetate dihydrate, (34 mm), tetraethylammonium hydroxide (TEAOH, 75 mm) and polyvinylpyrrolidone (PVP, 21.7 mm) were prepared. The precursor solution was prepared by mixing one volume unit of zinc acetate stock solution with one volume unit of PVP solution. Then one volume unit of TEAOH solution was added drop wise by use of a peristaltic pump under continuous stirring (flow rate 1.04 ml min^−1^). The reaction solution was obtained by adding 2 and 3 vol% nanopure water, respectively to the precursor solution resulting in a total volume of 1 ml. The functionalized substrates were subsequently immersed in the freshly prepared reaction solution and kept at 60 °C in an oil bath for 90 minutes. The wafers were thoroughly cleaned with methanol and dried under N_2_ stream. This mineralization cycle was repeated 20 and 40 times for all 2 vol% samples resulting in films with 002 texture and 15 times for the 3 vol% sample showing 100 texture.

### Characterization

X-ray diffraction (XRD) measurements were conducted on a Panalytical X’Pert MRD equipped with a Cobalt tube and polycapillary optics, an Eulerian cradle, a diffracted beam monochromator and a scintillation counter. Symmetrical *θ*–2*θ* scans were conducted covering the reflections 100, 002, and 101. Thereby, in order to avoid detection of diffracted intensity from the silicon substrate crystal the specimen was tilted using the cradle to an angle of *χ* = 10°.

The diffraction scans were evaluated by fitting a pseudo-Voigt function, considering the presence of the α_1_–α_2_ doublet with 2:1 intensity ratio, to each of the three recorded diffraction peaks as well as a background function. The ratio of the fitted integrated intensity values of the 002 and 100 reflections is taken as a measure for the preferred orientation of the ZnO crystallites. For a random powder, this ratio would be close to 1. Thereby, the tilting away from *χ* = 0° is ignored in the discussion.

Piezoresponse force microscopy (PFM) was performed on a Digital Instruments MultiMode^TM^ 8 from Bruker with a NanoScope V controller by applying an AC electric field (driving excitation) to the tip of the AFM while working in contact mode, and measuring the induced mechanical deformation of the sample at the same frequency due to the inverse piezoelectric effect. Stiff conductive tips with Pt coating were used (Bruker, MESP-RC). A standard sample of periodically poled lithium niobate (PPLN, Bruker) with known *d*_*33*_ was used as a reference. ZnO samples were glued on a holder via a conductive tab and were connected to the silicon substrate with silver paste. Driving excitations were applied with amplitudes ranging from 2 V to 10 V in 2 V steps and a frequency of 20 kHz to stay below the cantilever’s resonance frequency^28^. The first and second harmonic of the vertical piezoresponse signal were measured on five different places of each sample. For quantitative analysis, the vertical PFM amplitude values were averaged over the image’s area with the Nanoscope software and subsequently the averaged response on all areas for each applied bias was calculated. By determining the deflection sensitivity of the photodiode, the recorded mV signal was converted to a pm signal. The slope of the amplitude signal plotted over the applied bias (drive excitation) gives the *d*_*eff*_ value of the sample.

Detailed information on additional methods can be found in the Supplementary Methods: substrate preparation, purification of TMV, immobilization of single TMV, zeta potentials, SEM, and AFM.

## Additional Information

**How to cite this article**: Stitz, N. *et al*. Piezoelectric Templates – New Views on Biomineralization and Biomimetics. *Sci. Rep.* **6**, 26518; doi: 10.1038/srep26518 (2016).

## Supplementary Material

Supplementary Information

## Figures and Tables

**Figure 1:**
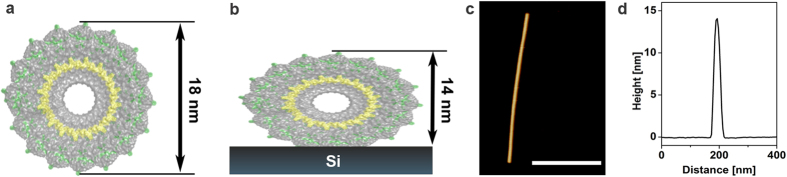
Deformation of Virus Cross-Section by Adhesion to Substrate. (**a,b**) Schemes of a TMV cross-section and its dimensions in solution (**a**) and immobilized on a hydrophilic oxidized silicon substrate (**b**) indicating the mechanical deformation of TMV due to strong adhesion via hydrogen bonding (obtained from pdb structure 3J06 manipulated with PyMol, the PyMOL Molecular Graphics System, Version 1.7.4 Schrödinger, LLC). The negatively charged RNA, which is incorporated in the TMV structure, is indicated in yellow. (**c,d**) Confirmation of the height decrease due to adhesion via AFM height image (**c**) and the corresponding height profile (**d**) revealing a height around 14 nm. The scale bar is 400 nm.

**Figure 2:**
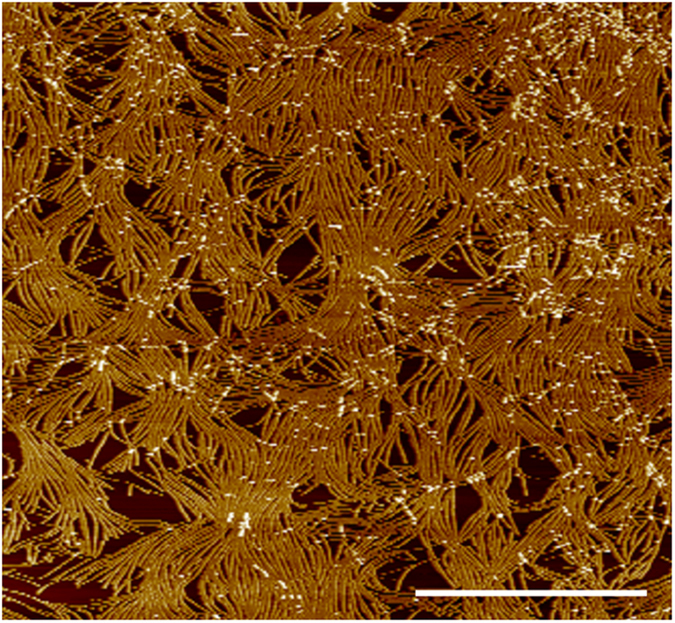
Formation of Homogeneous TMV Monolayers via Convective Assembly. AFM height image of a wtTMV monolayer prepared by convective assembly (*c* = 5 mg ml^−1^; withdrawal velocity *v*_*w*_ = 1.2 mm min^−1^; *V* = 5 μl). Withdrawal direction is from right to left. The scale bar represents 5 μm.

**Figure 3:**
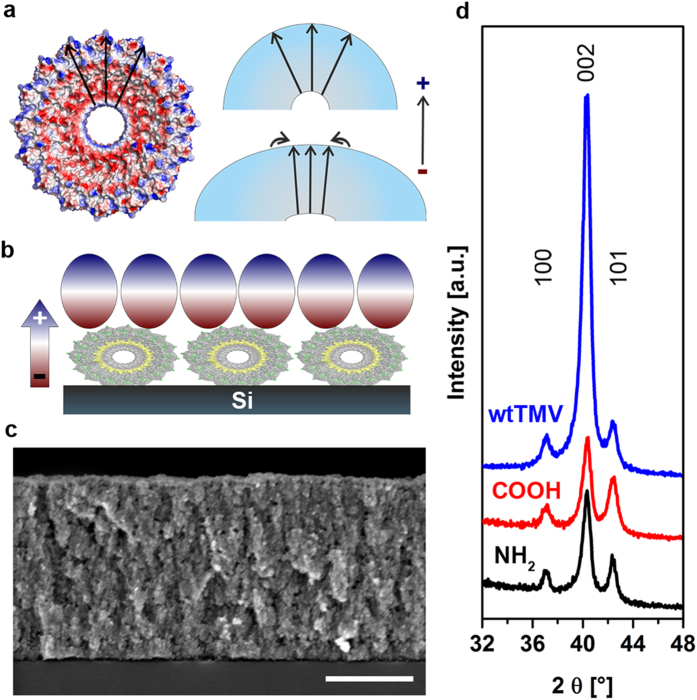
From Piezoelectric Template to Texture Enhancement. (**a**) Intrinsic dipole moment of TMV’s coat protein in the cross-section created with PyMol (left). The RNA was not taken into account in this visualization. Negative charges are indicated in red and positive charges in blue. The intrinsic dipole will further be enhanced under consideration of the negatively charged RNA. Visualization of the suggested rearrangement of the intrinsic dipoles due to mechanical deformation and the resulting parallel orientation of the dipoles when immobilized on a silicon substrate (right). (**b**) Condition for mineralization of wtTMV deformed on a silicon substrate. The piezoelectric effect occurring due to the mechanical deformation leads to a charged interface and also a flattened virus structure. Thus, only under this condition an optimum 002 texture can be obtained. (**c**) Cross-section SEM image of a wtTMV-based ZnO film after 40 mineralization cycles. (**d**) X-Ray diffraction (XRD) patterns of ZnO films prepared on NH_2_-SAMs, COOH-SAMs and wtTMV. In all cases, 002 textured ZnO is obtained. The strongly increased relative intensity of the 002 reflection of the wtTMV-based sample confirms the drastically enhanced 002 texture due to the piezo-electric effect upon deformation of wtTMV. The scale bar is 400 nm.

**Figure 4:**
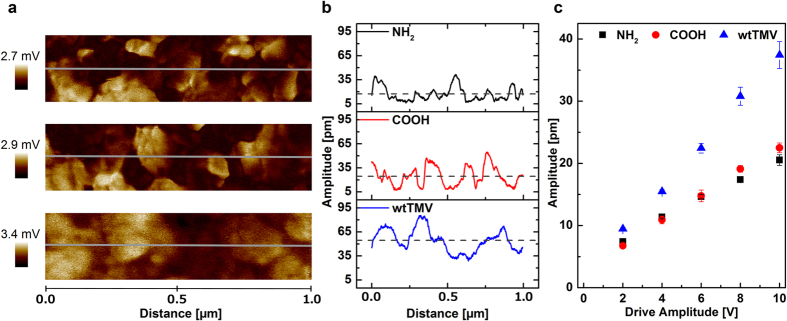
Piezoelectric Properties in Dependency of the Used Template. (**a**) Amplitude images of the PFM measurements performed at 10 V for NH_2_-based films (top), COOH-based films (middle) and TMV-based films (bottom). (**b**) Corresponding amplitude profiles taken along the grey lines in (**a**) are shown for each template. (**c**) Linear dependency of the vertical amplitude signal on the drive amplitude for each film. Data points correspond to mean values of five individual measurements. Error bars show the standard deviation from those mean values.
