# Supplementary material for: Piezoelectric Templates – New Views on Biomineralization and Biomimetics
Source: Sci Rep. 2016 May 23;6:26518. doi: 10.1038/srep26518 (PMC4876381; doi:10.1038/srep26518)
Supplement: Supplementary Information [file srep26518-s1.pdf]

Supplementary Information:

Piezoelectric Templates – New Views on Biomineralization and Biomimetics

Nina Stitz, Sabine Eiben, Petia Atanasova, Neus Domingo, Andreas Leineweber, Zaklina Burghard*, Joachim Bill

*corresponding author (email: zaklina.burghard@imw.uni-stuttgart.de)

Substrate preparation:

In this work, ZnO films were obtained via a template-assisted low-temperature chemical bath deposition method. Silicon wafers (100, *p*-doped polished wafers, Silchem, Germany), cut to 15 x 15 mm², were thoroughly cleaned by subsequent sonication in nanopure water and ethanol:acetone (1:1; v:v; 10 minutes each) followed by an oxygen plasma treatment (10 minutes, 30 W) and repeated sonication in nanopure water for 10 minutes. After each step the wafers were dried under an N_2 stream.

Purification of wtTMV:

Tobacco mosaic virus strain U1 was propagated in *Nicotiana tabacum* Samsun nn plants for 26 days. The infected leaves were harvested and the virus particles isolated according to the method of Gooding and Hebert [1]. In brief, the leaves were shred in a warring blender in sodium potassium phosphate buffer supplemented with mercapto ethanol. After removal of the cell debris, the virus particles were precipitated by two consecutive PEG6000 precipitation steps. For final purification the resuspended virus particles were pelleted by ultracentrifugation. The virus particles were then resuspended in nanopure water and stored in the fridge.

Single immobilized viruses

Single virus particles were immobilized on silicon substrates to determine the height of wtTMV via atomic force microscopy (AFM). Freshly cleaned silicon substrates were incubated with a buffer-free wtTMV solution (3 µL, 0.2 mg mL^{-1}) for 10 minutes. After removal of excess solution, the substrates with the immobilized viruses were dried under N_2 stream and analyzed via AFM (see details in Section Characterization – AFM). To measure the additional height reduction due to treatment with the reaction solution, immobilized wtTMV on silicon were immersed in pure methanol at 60°C for different time spans between 5 and 15 minutes. Pure methanol was chosen to avoid any instant mineralization usually occurring directly after immersion in the reaction solution. Substrates were removed from and washed with methanol several times. After drying under an N_2 stream the height was determined via AFM.

Characterization:

To determine the film thickness, scanning electron microscopy (SEM) a field emission SEM Zeiss DSM982 Gemini with a thermal Schottky field emitter was performed. The microscope was operated at 3 kV acceleration voltage and a working distance of 3 mm. Samples were broken and glued on cross-section SEM holders with carbon conductive tabs. The samples were covered by sputter deposition of 0.5 nm Pt:Pd (80:20) alloy.

Atomic force microscopy (AFM) was performed on a Digital Instruments MultiMode™ 8 from Bruker with a NanoScope V controller operated in tapping mode to measure the reduced virus height of single viruses due to interaction with the hydrophilic oxidized silicon substrate and the coverage of the convectively assembled wtTMV monolayers. The height of single immobilized wtTMV particles was determined from the measured height images. Therefore, the image background was flattened with the Nanoscope software according to the manufacturer's

information (Bruker). Subsequently, the height was averaged over the length of the virus and exported as a plot. PPP-NCHR-W (Nanosensors) n^+ doped silicon tips with resistivity 0.01 - 0.02 Ω cm were used.

To determine the zeta potential of wtTMV at different pH values, the particles were first precipitated by ultracentrifugation (Beckman Coulter Optima L-90 k, 45 Ti Rotor) at 34000 rpm for 115 minutes at 4°C. The pellets were resuspended overnight in 1 mM NaCl at pH 5.5 and 1 mM NaCl at pH 8, respectively, to a final concentration of 0.5 mg mL^{-1}. The pH was adjusted with NaOH. Measurements were performed with a ZetaSizer Nano ZS in DTS 1070 Cells at 25°C. Three individual measurements with automatic subruns were performed for both pH values. The zeta potential was evaluated according to the Smoluchowski equation. The given values are the average values of the three individual measurements and their standard deviations.

The zeta potentials of the COOH- and NH_2-SAMs were determined on a SurPASS Electrokinetic Analyzer (Anton Paar GmbH). COOH- and NH_2-functionalized silicon substrates (10 x 20 mm²) were glued with double sided tape on the stamps of the variable gap cell. The gap height was adjusted to 100 µm. A 1 mM KCl buffer solution was purged with N_2 prior and during the measurements. Automatic titration was conducted with a 0.1 M HCl solution. Four pressure ramps from 0 to 400 mbar were applied for each data point and the streaming current was measured. The zeta potential was calculated with a Fairbrother-Mastin approach.

Texture:

X-ray diffraction (XRD) revealed the formation of ZnO 002 textured films for all templates, which is indicated by the increased relative intensity of the 002 reflection compared to the 100 and 101 reflection (Figure 3d). However, the degree of orientation differs. Whereas both, ZnO growth on COOH- (ZnO_{002}/COOH) and NH_2-terminated SAMs (ZnO_{002}/NH_2), show comparable

results, the texture of the TMV-based ZnO film (ZnO_{002}/TMV) is drastically increased, which we attribute to the induced piezoelectric effect of the deformed TMV. To quantify this effect, the texture coefficient T was calculated as the ratio of the integrated intensities of the 002 and the 100 reflections. The resulting values are $T_{ZnO/TMV} = 12.7$, $T_{ZnO/NH_2} = 9$ and $T_{ZnO/COOH} = 5.9$.

Zeta potential:

Investigations of the zeta potential ζ of, on the one hand, wtTMV particles in solution and, on the other hand, on COOH- and NH_2-functionalized silicon substrates were performed (Figure S1). The measurements were conducted at pH 5.5 and at pH 8 which is close to the mineralization conditions. NH_2-SAMs have a slightly positive zeta potential ζ at pH 5.5 and a more negative zeta potential ζ at pH 8.

Both wtTMV and COOH-SAMs are negatively charged at the given pH values. However, the absolute values are nearly doubled in the case of the COOH-SAMs compared to wtTMV reaching values of -99 ± 1mV compared to -53 ± 1mV at pH 8.

If the deposition was electrostatically controlled, the template with the largest absolute zeta potential ζ would show the highest degree of texture. However, as can be seen in Figure S 2, the highest absolute ζ potential was obtained with COOH-SAMs followed by wtTMV particles and the lowest absolute value for NH_2-SAMs. Since the highest degree of texture is obtained on wtTMV (cf. Figure 3d), electrostatic contributions to the deposition can be ruled out.

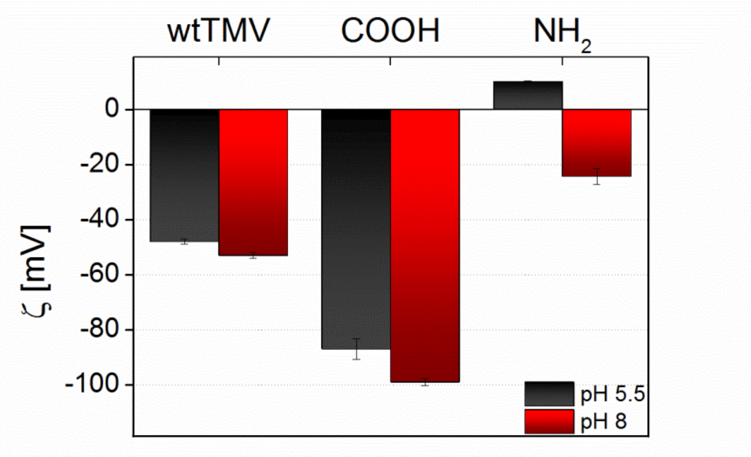

Figure S1: Plot of the zeta potential of wtTMV particles, COOH, and NH2-functionalized silicon substrates at pH 5.5 and pH 8.

Within the methanol reaction solution the dipole/dipole interactions of piezoelectric TMV and polar ZnO might dominate deposition. In solvents with low dielectric constants, such as methanol ($\varepsilon = 33.6$) the capability of a solvent to electrolytically dissociate functional groups is strongly reduced. Consequently, neither the COOH- and NH_2-SAMs nor the TMV particles are expected to be charged in the methanol solution and the deposition is not driven by electrostatic interactions. Zeta potential measurements performed on the SAMs as well as the single TMV particles supported the lack of electrostatic contributions to the mineralization process.

PFM on wtTMV-based sample with 40 cycles

PFM measurements were also performed on samples with a higher thickness. The results for wtTMV after 40 applied mineralization cycles are shown in Figure S2. However, measuring thick films by PFM leads to problems caused by the measurements set-up and the resulting inhomogeneous electric field. The d_{33} coefficient is defined considering that the piezoelectric response is measured using a capacitor-like structure, which would imply immersion of the material in homogeneous electrical field. However, in the case of the PFM measurements the

field emerging from the tip is rather a point charge than a planar field [4]. Thus, the effective voltage sensed by the material will be smaller for a thicker sample due to the more radial electric field over the volume leading to a smaller effective d_{33}. This is supported by our results. The d_{eff} value of the wtTMV sample decreases from 3.8 pmV^{-1} (20 mineralization cycles) to 3.4 pmV^{-1} (40 mineralization cycles). Thus, all PFM measurements were performed with samples mineralized applying 20 deposition cycles.

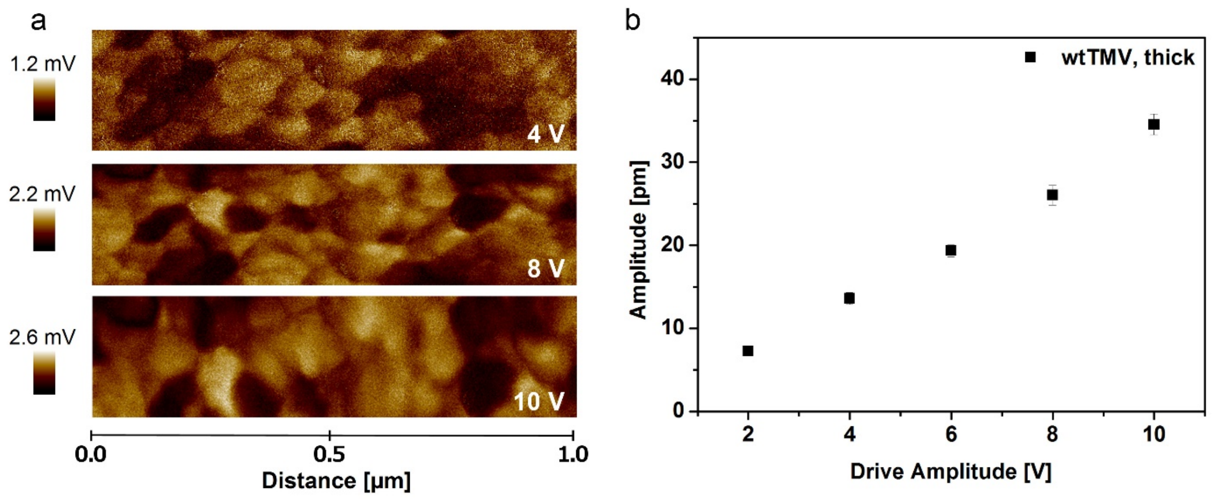

Figure S2: a, PFM amplitude images of a thick ZnO layer on wtTMV (40 mineralization cycles) at 4 V, 8 V and 10 V drive amplitude and b, piezoresponse plotted over the applied bias obtained from 5 individual images for each bias and averaged amplitude over the image's area. The resulting d_{eff} is 3.4 pmV^{-1}.

100 versus 002 texture

The influence of the crystal orientation on the ZnO piezoelectric properties was evaluated by studying homogeneous smooth 300 nm thin ZnO/NH_2 films in 100 and 002 orientations prepared by adjusting the reaction solution (Figure S3a and b). XRD data prove that ZnO_{002}/NH_2 exhibits

only a small 002 texture (Figure 3d) whereas the ZnO_{100}/NH_2 has a 100 texture (Figure S3d) as indicated by the changed ratios of the relative intensities of the reflections.

In Figure S3e and f) the vertical electromechanical response (amplitude PFM image) is given for both samples for driving excitations of 4 V and 8 V, respectively. Areas in the amplitude PFM images with brighter contrast indicate areas with higher electromechanical response. Very dark areas show a weak response. The ZnO_{002}/NH_2 sample shows several domains with varying response due to a varying *c*-axis orientation of the crystallites, as already indicated by XRD. The overall response was increased by doubling the driving excitation, and the image has a better signal to noise ratio (Figure S3e). Subsequently, the drive excitation was varied in 2 V steps from 2 to 10 V in order to determine the piezoresponse effective constant d_{eff} of the samples (Figure S3g). The piezoresponse of ZnO_{002}/NH_2 increases linearly with increasing applied voltage indicating good electromechanical behavior. The d_{eff} value is in the region of 1.6 pm V^{-1} which in our case can lead to an estimation of d_{33} = 3.2 pm V^{-1}. In contrast, the amplitude for ZnO_{100}/NH_2 stays constant with increasing bias. The measured signal is in the region of the system inherent noise level. The sample shows, as expected, no piezoelectric behavior since only the 002 texture ZnO orientation shows piezoresponse. However, the achieved d_{33} of ZnO_{002}/NH_2 is low and only slightly higher than the signal on bare silicon (Figure S4) due to poor 002 texture on nonpiezoelectric template.

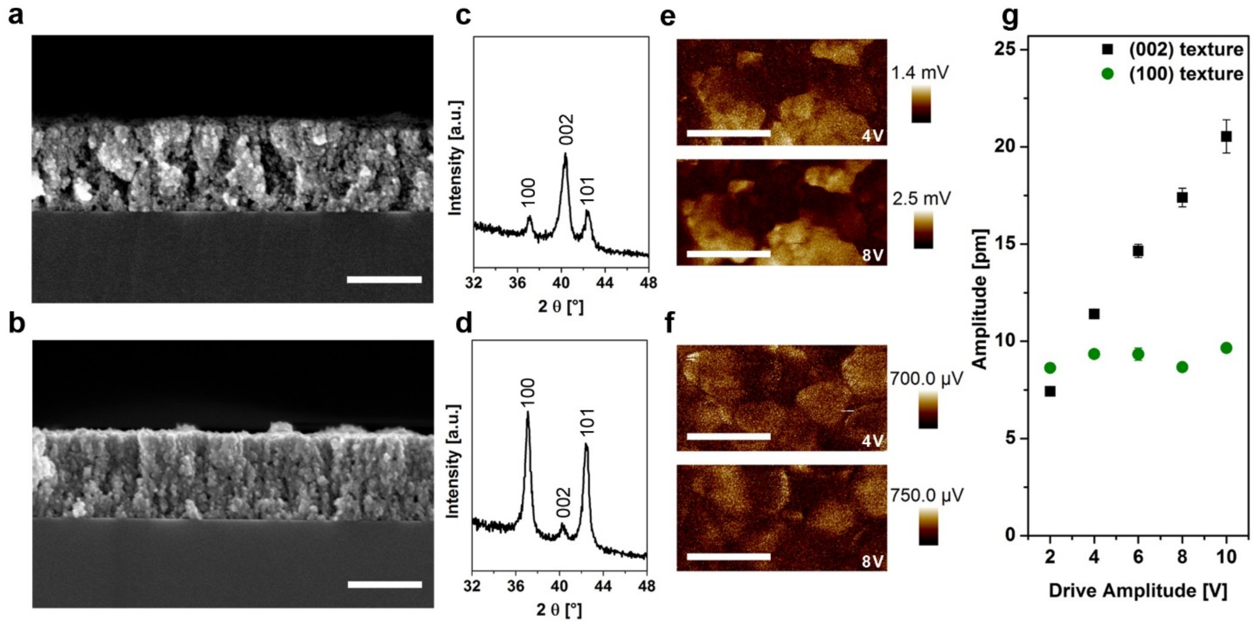

Figure S3: a,b, Cross-section SEM images of NH_2-based homogeneous smooth ZnO thin films with small degree of 002 texture (a) and more pronounced 100 texture (b). c,d, XRD patterns of the films confirm ZnO in wurtzite structure in both cases with different texture. The first film shows only a small 002 texture which is proven by the slightly increased relative intensity of the 002 reflex (c) whereas, a 100 texture (d) is confirmed for the second film by the increased relative intensity of the 100 reflection. e,f, Corresponding amplitude images of PFM measurements at 4 V and 8 V for films with slight 002 texture (e) and 100 texture (f). g, Vertical response plotted in dependency of the applied drive amplitude. Data points represent five individual experiments. Error bars show the corresponding standard deviation of the mean value. All scale bars are 250 nm.

PFM on bare silicon

PFM measurements were also performed on a bare silicon wafer with native SiO_2 layer (cleaned in the same way) as a reference. The sample did not show any amplitude or phase contrast at all applied biases (Figure S4a). The amplitude signal was also averaged over the image area and

plotted over the drive amplitude (Figure S3b). A slight increase of the signal can be observed corresponding to a d_{eff} of 0.6 pmV^{-1}.

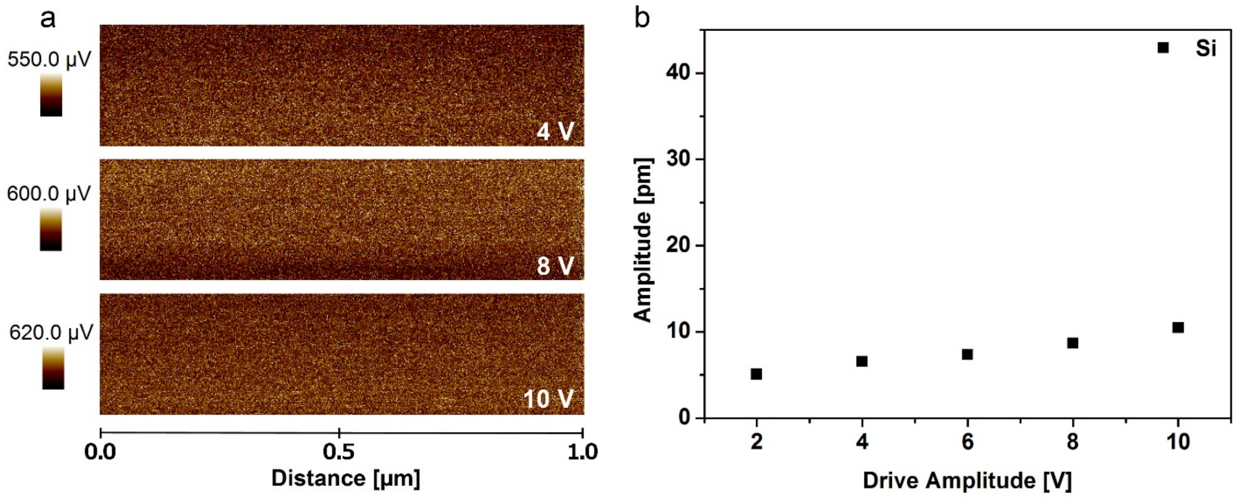

Figure S4: a, PFM amplitude images of a bare silicon wafer with SiO_2 top layer at 4 V, 8 V and 10 V drive amplitude and b, piezoresponse plotted over the applied bias obtained from 5 individual images for each bias and averaged amplitude over the image's area. The resulting d_{eff} was determined to be 0.6 pm V^{-1}.

References

1. Gooding, G. V. & Hebert, T. T. A Simple Technique for Purification of Tobacco Mosaic Virus in Large Quantities. *Phythopathology* 57, 1285-& (1967).

2. Schwer, C. & Kenndler, E. Electrophoresis in fused-silica capillaries: the influence of organic solvents on the electroosmotic velocity and the .zeta. potential. *Anal. Chem.* 63, 1801–1807 (1991).

3. Rived, F. Rosés, M. & Bosch, E. Dissociation constants of neutral and charged acids in methyl alcohol. The acid strength resolution. *Anal. Chim. Acta.* 374, 309–324 (1998).

4. Christman, J. A. Woolcott, R. R. Kingon, A. I. & Nemanich, R. J. Piezoelectric measurements with atomic force microscopy. *Appl. Phys. Lett.* 73, 3851 (1998).
